# Efficacy of Nx4 to Reduce Plasma Cortisol and Gastrin Levels in Norwegian Sled Dogs During an Exercise Induced Stress Response: A Prospective, Randomized, Double Blinded, Placebo-Controlled Cohort Study

**DOI:** 10.3389/fvets.2021.741459

**Published:** 2021-10-26

**Authors:** Alexandra Keller, Jana Conradi, Corinna Weber, Klaus Failing, Melanie Wergin

**Affiliations:** ^1^Tierärztliches Orthopaedie Team Frankfurt, Frankfurt, Germany; ^2^Dartsch Scientific GmbH, Wagenfeld, Germany; ^3^Laboklin GmbH & Co. KG, Bad Kissingen, Germany; ^4^Unit for Biomathematics and Data Processing of the Veterinary Faculty, University Giessen, Giessen, Germany; ^5^Biologische Heilmittel Heel GmbH, Baden-Baden, Germany

**Keywords:** dog, exercise-induced stress, HPA, blood cortisol, gastritis, Nx4

## Abstract

**Introduction:** An exercise induced stress response is commonly seen in high performance sled dogs, resulting in increased plasma cortisol. A stress induced rise of cortisol might result in increased prevalence of gastritis and gastric ulcers mediated by an increase of gastrin. Neurexan^®^ (Nx4) is a medicinal product used for stress relief by reduction of cortisol. The aim of the study was to show that Nx4 reduces plasma cortisol and plasma gastrin in high performance sled dogs and to show tolerability of Nx4 in dogs.

**Material and Methods:** First, a pilot study was done to validate the increase of cortisol by performance. The data from the pilot study was used for sample size estimation via an adapted power analysis as well as the identification of important variables. These were then used in the randomization procedure of the main study. Second, a prospective randomized, double blinded, placebo-controlled cohort study was conducted. The main study included 45 sled dogs, assigning 23 dogs to the Nx4 group, and 22 dogs to the placebo group, to analyze plasma cortisol and plasma gastrin at four time points: before, directly after and 30 and 120 min after performance.

**Results:** For the main target variable, area under the curve (AUC) of plasma cortisol, a significantly lower adjusted mean value in the Nx4 group compared to the placebo group (*p* = 0.031) was found. Plasma gastrin was also significantly reduced in the Nx4 group 30 min after performance (*p* = 0.023), resulting in a significantly reduced plasma gastrin AUC in the Nx4 group compared to the placebo group (*p* = 0.049).

**Discussion:** Within the limitation of the study, the results carry implications for the usefulness of Nx4 to reduce exercise induced plasma cortisol and gastrin levels. The reduction of the exercise induced stress response could help to improve the welfare of high-performance sled dogs. Since activation of the hypothalamic-pituitary-adrenal axis resulting in increased cortisol is similar for exercise induced stress and psychologic stress, the same might be true independent of the stressor, making Nx4 potentially useful in any stressful situation for dogs.

## Introduction

An exercise induced stress response with increased blood cortisol levels is commonly seen in sled dogs (1–3). Exercise represents a physical stress, challenging homoeostasis of the body, resulting in a physiological response (4). The primary factor determining the endocrine stress response to a single acute session of exercise, is the intensity, or duration of exercise. If the exercise is reaching a certain threshold, there is an increase in numerous stress-related hormones, such as Adrenocorticotropic hormone (ACTH), epinephrine, nor-epinephrine, and cortisol is induced (5, 6). This study is focusing on cortisol as a biomarker for the exercise induced stress response, since ACTH, nor-epinephrine, and epinephrine are of very short half-life and are difficult to measure in a clinical setting (7). One of the key mediator of the exercise induced stress response is the activation of the hypothalamic-pituitary-adrenal axis (HPA axis) (6, 8).

The neuroendocrine stress response to a single acute exercise session is of short duration, with cortisol levels returning rapidly to baseline (9). Exercise related stress is inducing either adaptive or maladaptive changes in the homeostasis of the body, activating catecholamines, cortisol, and certain cytokines. If an exercise induced stress stimulus is repetitive, the recovery to the homeostatic levels may be incomplete. As a result, the body can define a new, increased set point of basal values, for example for cortisol. The difference between the new and the old set points can be understood as an adaptation to stress (10, 11).

A stress induced rise of cortisol can result in increased prevalence of gastritis and gastric ulcers (12, 13). Gastrin is a potent hormone, stimulating gastric acid and pepsin secretion. Under physiological conditions, gastrin secretion is mainly mediated by gastrin releasing peptide (GRP). Gastric acid hypersecretion plays an important role in the pathogenesis of stress ulcers (14). Gastritis and gastric ulcers are common in racing sled dogs with a prevalence of 41.5–61% (15–17) and significantly increased cortisol and gastrin levels were found in sled dogs after endurance racing and recreational mushing (1, 18).

Neurexan^®^ (Nx4) is used in human medicine for stress relief and sleep-induction. Nx4 is a medicinal product, consisting of three herbal extracts (Avena sativa, Coffea arabica, Passiflora incarnata) and one mineral salt (Zincum isovalerianicum) in low but measurable concentrations. Nx4 has been extensively investigated within the last years (19–24) showing a significant reduction of nervousness/restlessness in patients (21). Also a significant reduction of salivary cortisol and plasma adrenaline levels, as response to an acute stressor in healthy volunteers, has been demonstrated with Nx4 (20). In an experimental setting, significant changes in delta- and theta-waves of the electro-encephalogram (EEG) of rats have been observed, being indicative for a calming effect of Nx4 (19). Also in healthy volunteers an alteration of the EEG after Nx4 treatment has been found (24). It was recently demonstrated by functional magnetic resonance imaging (fMRI) that Nx4 significantly reduced the activation of the amygdala and other stress-relevant brain areas under stress conditions, shown. The amygdala plays a crucial role in mediating the body's response to stress (23).

We hypothesized that plasma cortisol and plasma gastrin are significantly increased by performance in Norwegian sled dogs. The first aim of the study was to show that Nx4 reduces plasma cortisol and plasma gastrin. The second aim was to show that Nx4 is well-tolerated in dogs.

## Materials and Methods

### Ethic Statement

Both study parts were approved by the Norwegian Food and Safety Authority (FOTS ID 17660) based on the use of animals for scientific purposes (forskrift 18. juni 2015 nr. 781om bruk av dyr i forsøk). The owners gave informed, written consent for the inclusion of their dogs in this study.

### Sled Dogs

Privately owned Siberian Huskies were entered into the study. A population of interrelated dogs was utilized as to provide a more homogenous population for study purposes.

All dogs participating in the study received the dry food VIP extreme (V.I.P. dogfood, Frogner, Norway) containing salmon, chicken and krill (5%), rice, sage, psyllium, glucosamine (800 mg/kg) and chondroitine (500 mg/kg), L-carnitine (300 mg/kg), and lecithin (250 mg/kg), containing 4,800 Kcal/kg. The guaranteed analysis of the feed was: protein 32%, fats 26%, fiber 2%, ash 7.5%, calcium 1.6%, phosphorus 1%. Feed additives per kg: 20.000 IE Vit. A, 1.800 IE Vit. D3, 500 U.I/kg Vit. E (DLA- Tocoferolacetat), 250 mg iron (ironsulfat, monohydrate), 200 mg/kg Vit. C, 25 mg/kg copper, 60 mg/kg manganese, 200 mg zinc, 2.5 mg iodine, 0.5 mg selenium, 1.200 mg/kg taurine. The amount of dry food per day deemed the necessary amount to maintain a racing body condition score (BCS) of 4–5. Dogs were fed twice daily.

### Clinical Examination

A brief clinical and orthopedic examination included the history of the dog reported by the owner, observation of the dog standing and moving, as well as palpation of the dog. The dogs weight as well as the BCS was recorded using the Purina BCS with a nine-point scale (25). The ideal BCS for dogs competing in mid-distance races is 4–5 (25, 26).

Norwegian sled dogs of either gender and with at least 2 years of age were eligible for inclusion. All dogs were required to be healthy based on clinical examination and owner perception.

Dogs showing any signs of discomfort during standing, moving, or having pain on palpation were not eligible for the study. Dogs receiving any treatment within 14 days before enrollment were excluded from the study. A dog with a BCS of <3/9 was not eligible for the study.

### Blood Sample Collection

All blood samples (5–6 ml per sample) were taken from the vena jugularis externa with a 20-gauge needle. The blood was taken as fast as possible (within 10 min for all dogs in one team) and the blood was processed immediately. At the first time point, blood was transferred to an EDTA tube for complete blood count (CBC) (in the main study) and into a lithium heparin tube to obtain plasma. Four blood samples were collected from each dog: first sample before exercise (baseline value), second sample directly after exercise as well as 30 and 120 min thereafter. The lithium heparin tube was centrifuged immediately for 10 min at 2,000 × g and the plasma was stored in two labeled aliquots at −20°C until departure. At the end of the study the samples were transported on dry ice to the laboratory for analysis.

In the pilot study EDTA blood for whole blood analysis was taken on the last day and was transferred to the laboratory within 24 h. For higher accuracy, in the main study the CBC was analyzed immediately at the study site with a scil Vet abc Plus+ (Scil GmbH, Viernheim, Germany).

### Laboratory

Clinical chemistry testing including alkaline phosphatase (AP), aspartate aminotransferase (AST), gamma-glutamyl transpeptidase (GGT), glutamatdehydrogenase (GLDH), DGGR- Lipase, total bilirubin, cholesterol, triglyceride, total protein, albumin, globulin, glucose, fructosamine, uric acid, creatinine, calcium, sodium, potassium, magnesium, anorganic phosphate, iron, creatine kinase (CK), and lactate was done photometrically with a Cobas8000 c701 modulated analyser (Roche GmbH, Mannheim, Germany). Plasma cortisol was analyzed with an electrochemiluminescence immunoassay using biotyinylated sheep monoclonal anti-cortisol antibodies and synthetic ruthenylated cortisol hapten on a Cobas8000 e602 modulated analyzer (Roche GmbH, Mannheim, Germany) including two internal controls. The cortisol test was validated for canine samples, with intra- and inter-assay variation coefficients of 1.5–5.4 and 1.9–10.1%. respectively. The lab is regularly and successful taking part in external quality assurance schemes for cortisol in canine samples, offered by the European Society of Veterinary Endocrinology (ESVE) and the test was used in a previous clinical study (27).

Gastrin was measured using a chemiluminescent enzyme-labeled immunometric assay with murine monoclonal antibodies against gastrin as capture antibodies as well as an anti-ligand-coated solid phase. The test was validated for canine samples and was used in a previous clinical study (28).The test was performed on an Immulite 2000 XPi (Siemens Healthcare GmbH, Erlangen, Germany) analyzer with two synthetic human G-17 gastrin internal control samples. The intra- and inter-assay variation coefficients were 3.2–6.2 and 4.7–9.8%, respectively.

### Intervention

The tested drug was Neurexan^®^ (Nx4) manufactured by Heel GmbH (Nx4 tablets: lot No. 88972, manufacturing date 08.08.2019; placebo tablets: lot No. 88923, manufacturing date 01.08.2019).

Nx4 is a medicinal product in tablet form and manufactured according to the German Regulation and international GMP standards. Ingredients of Nx4 are Passiflora incarnata (purple passionflower) D2 0.6 mg, Avena sativa (common oats) D2 0.6 mg, Coffea arabica (coffee) D12 0.6 mg, and Zincum isovalerianicum (valerianate of zinc) D4 0.6 mg. In addition, carriers incorporated in the tablets are small amounts of lactose monohydrate and magnesium stearate and were the only constituents used in the placebo tablets. Nx4 and placebo were identical in terms of taste, size, color, and labeling.

### Statistics

Statistical analyses were performed using the statistical program package BMDP. For nearly normal distributed variables, the data description was given by the arithmetic mean and standard deviation (SD). Graphical representation of the data was given by box-and-whisker-plots in all cases. Due to the right-skewed distribution form of the hormone values and the liver enzymes, these variables were transformed logarithmically before statistical analysis.

For the variables with repeated measures (creatinine kinase, plasma cortisol, lactate, gastrin, plasma GRP) the area under the curve (AUC) was computed to consider the complete observation period in one step and further to reduce the dimension of the analysis. Calculating the AUC with 3 time points can increase the variance but this was taken into account in the statistical analysis. The advantage of the AUC is that the initial values can be considered as a covariate taking the significant impact of the baseline value into account. For the variable plasma gastrin values below the detection limit of 10 ng/ml were replaced by the fictive expectation value of 7 ng/ml.

For variables with only one observation over time, the mean comparison between the treatment groups was done with the *t*-test for independent samples or the Wilcoxon-Mann-Whitney-test (program BMDP3D). For the variables which are possibly dependent from their basal value (esp. most AUC's), a one-way analysis of covariance (ANCOVA) with the basal value as a covariate was performed for the comparison of the adjusted means and to obtain a higher accuracy, respectively, power of the comparisons. Additionally, the slopes of the dependency were compared between the groups to test their homogeneity (program BMDP1V). A three-way ANOVA (BBW) mixed model was done to evaluate the random effect of dog (age, weight, and BCS) and the effect of the musher. As an alternative to this analysis a modified AUC form was computed, subtracting the basal value from the further values, and then building the AUC. In this case, the mean comparison could be performed by the *t*-test (program BMDP3D). For comparison of multiple time points, repeated measures ANOVA was used.

For all variables, the common level of statistical significance was set at *P* ≤ 0.05. In this context, the variables plasma cortisol and plasma gastrin were considered as the main target variables.

### Pilot Study

The pilot study was designed as an observational study. The data from the pilot study was used for sample size estimation via an adapted power analysis as well as the identification of important prognostic variables to be considered for randomization of the main study. Eighteen dogs were screened for inclusion in the pilot study. One dog was scared by handling and blood taking, so the dog was excluded from the study.

A total of 17 dogs were included in the pilot study in November 2018 at the beginning of the training season, starting after the summer break. The dogs had no training during the summer due to the high temperatures. All dogs in the pilot study were from the same musher. Dogs had the same routine of feeding twice a day and training every other day during the training season.

On the first day, all equipment was installed, and the musher was informed about the exact study course. One team per day was measured with 6 dogs per team (the dog that dropped out of the study was allowed to run). Each team ran once during the study. Each team left between 11:00 and 12:00. The distance for each team was 25 ± 2 km within 140 ± 15 min. All teams used the same route to control for distance, speed, and steepness of the trail.

From the 17 participating dogs CBC and the biochemistry profile was measured at baseline. Lactate, CK, plasma cortisol, and plasma gastrin were analyzed before (baseline values), directly after, as well as 30 and 120 min after the training session.

### Main Study With Nx4

The study was designed as a prospective, randomized, double blinded, placebo-controlled cohort study. The main study was conducted in the middle of the training season in 2019. Forty-six dogs were screened, and 45 dogs were entered in the main study. The number of dogs assigned to this part of the study was based on a power analysis, with a power of 80% at an alpha level of 0.05 and with the addition of 10% of the calculated number to control for drop-outs after screening. One dog dropped out 1 month after screening before entering the study due to back problems, leaving 45 dogs in the study with 23 dogs in the Nx4 group and 22 dogs receiving placebo. Dogs were randomized based on gender, age, and plasma cortisol. The baseline plasma cortisol (plasma cortisol before exercise) for randomization was obtained from the pilot experiment for 17 dogs. In September 2019 additional 29 dogs were recruited for the main study. From these dogs, blood was taken to obtain baseline plasma cortisol value (baseline plasma cortisol without exercise) for randomization, as well as CBC and blood chemistry to assure overall health of the dogs before participation in the study.

The time schedule for the main study was similar on each day for all teams. The time schedule for the main study started with waking up the dogs at 6:30 by the musher to assure that all dogs were fine. The dogs were fed at 7:00, and at 8:00 all dogs participating on that day received a health check and the microchip was read for identification. All dogs were clearly identifiable by a collar with the name of the dog or a name tag, attached securely to the collar to assure that dogs received the correct medication. At that time point the first blood sample was taken (baseline values, before exercise). CBC and the biochemistry profile were measured at baseline before exercise. Lactate, CK, plasma cortisol, and plasma gastrin were analyzed before (baseline values), directly after, as well as 30 and 120 min after the training session.

Beginning at 8:30 all dogs received 2 tablets of either Nx4 or Placebo every 30 min for five times, that makes a total of 10 tablets per dog before exercise.

All study personnel as well as the mushers were blinded to the study treatment during the study. Nx4 and placebo were identical in terms of taste, size, color, and labeling; both contained lactose monohydrate and magnesium stearate. The investigator kept the treatment code envelopes throughout the course of the study locked and was not authorized to break the code without a valid reason (e.g., in case of emergency). There was no emergency unblinding in this study.

Approximately at 10:30 the quad left. The distance for each team was 35 ± 1.2 km within 180 ± 9 min. The dogs belonged to 3 mushers, and dogs were grouped accordingly into five teams. Mushers participated only with their own dogs. Each team included dogs from the Nx4 and the placebo group.

## Results

### Pilot Study

Clinical and orthopedic examinations were within normal limits for all participating dogs as well as CBC and biochemical blood analysis. The pilot study included 10 intact males and 7 intact females with a mean age of 6.2 years (range: 3–11 years).

Plasma cortisol and plasma gastrin before exercise were not significantly different in males and females (mean basal cortisol female: 22.16 ng/ml, mean basal cortisol male: 18.0 ng/ml; *p* = 0.5). Neither had age (*r*^2^ = 0.36), and BCS (*r*^2^ = 0.0086) an influence on baseline plasma cortisol (before exercise) or baseline plasma gastrin [mean basal gastrin female: 7.8 pg/ml, mean basal gastrin male: 8.3 pg/ml; *p* = 0.70; *r*^2^(age) = 0.0003; *r*^2^(BCS) = 0.001] concentration in this cohort of dogs (Table 1). Dogs did not show signs of gastritis based on clinical examination and owner perception.

**Table 1:** Plasma cortisol and plasma Gastrin levels of the pilot study.

**Parameter**	**All dogs** **(***n*** = 17)**	***P*** **-value#**	**Male** **(***n*** = 10)**	**Female** **(***n*** = 7)**	***P*** **-value##**
**Plasma cortisol (ng/ml, mean ± SD)**					
Before exercise	19.7 ± 14.0	–	18.0 ± 4.5	22.16 ± 5.4	0.5
After exercise	73.9 ± 30.2	0.001	69.4 ± 9.7	80.3 ± 11.6	0.5
30 min after	55.4 ± 24.2	0.001	53.8 ± 7.9	57.8 ± 9.4	0.8
120 min after	33.7 ± 16.0	0.007	36.5 ± 5.1	29.8 ± 6.1	0.4
**Plasma Gastrin (pg/ml, mean ± SD)**					
Before exercise	8.0 ± 2.4	–	7.8 ± 0.8	8.3 ± 1.0	0.7
After exercise	9.4 ± 3.9	0.14	9.4 ± 1.3	9.5 ± 1.5	0.9
30 min after	8.6 ± 3.4	0.44	9.4 ± 1.1	7.6 ± 1.3	0.3
120 min after	9.0 ± 3.5	0.33	9.0 ± 1.2	9.0 ± 1.4	0.9

*SD, standard deviation*.

# *comparing plasma Cortisol at each time point to before exercise values*.

## *comparing male vs. female*.

Baseline cortisol levels correlated to baseline alkaline phosphatase (ALP) (*r*^2^ = 0.55, *p* = 0.0006), showing the influence of cortisol on the secretion of ALP (2, 29).

**Figure 1:**
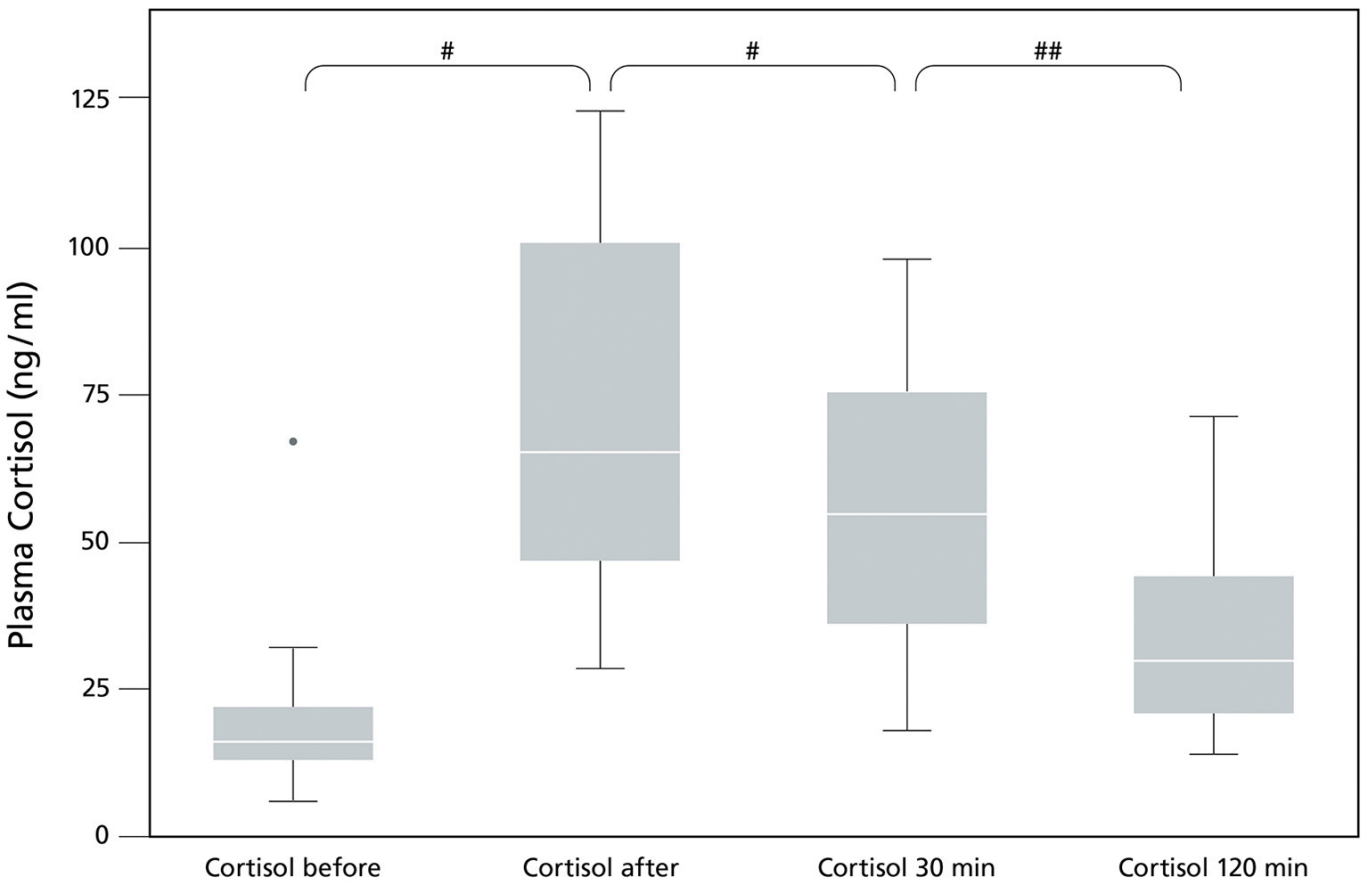
Plasma Cortisol levels (ng/ml) in sled dogs before and after endurance exercise (pilot study). The data (*n* = 17) are displayed as boxplot showing the first (25%), the median (50%) and the third quartile (75%). The whiskers are displaying values within 1.5 times the interquartile range (IQR). Outliers are shown separately. Outliers are plotted as individual points. ^#^*p* < 0.001, ^##^*p* = 0.013.

Plasma cortisol increased significantly directly after performance (*p* = 0.001), followed by a significant decrease of plasma cortisol 30 min after performance (*p* = 0.001). Plasma cortisol levels went back slightly above baseline at the 120 min timepoint (*p* = 0.013) (Figure 1; Table 1) showing a significant effect of time (*p* = 0.0001).

To assess the strenuousness of the exercise, lactate and creatine kinase were measured before and after performance. Creatine kinase increased significantly (*p* = 0.026), not reaching baseline within 120 min after performance (mean CK before: 47.0 ± 33.5 U/L; mean CK after: 134.1 ± 148 U/L; mean CK 120 min after: 116.8 ± 63.1 U/L). Lactate increased significantly (*p* = 0.015) during the exercise reaching values below baseline 120 min after performance (mean lactate before: 1.7 ± 0.7 mmol/L, mean lactate after: 2.2 ± 0.5 mmol/L; mean lactate 120 min after: 0.9 ± 0.2 mmol/L).

### Main Study With Nx4

The main study was conducted in the middle of the training season in 2019. The time from the pilot study (November 2018) to the main study was needed for data analysis and the recruitment of additional dogs. In addition, due to the observed low gastrin levels in the pilot study, the study was done in the middle of the training season.

The main study included 26 intact males and 19 intact females, with 14 male and 9 females in the Nx4 group and 12 male and 10 females in the placebo group. There was no significant mean difference between groups at baseline (before exercise) for age, BCS, gender, plasma cortisol, plasma gastrin, plasma lactate, leucocyte count, or hemoglobin. Dogs did not have significantly different plasma cortisol levels in mean values, in dependence of the musher or the team. Additionally, there are no statistically significant three-way interactions in a Three-way ANOVA (BBW) mixed model analysis for any of the parameters tested (gender, age, BCS, and musher).

Demographic data are displayed in Table 2, raw data are given in Supplementary Table 1.

**Table 2:** Demographic data of the main study.

**Characteristic**	**Nx4 group (***n*** = 23)**	**Placebo group (***n*** = 22)**	***P*** **-value for difference between groups**
Age (years) mean ± SD	4.1 ± 2.2	4.4 ± 2.2	0.68
BCS mean ± SD	4.5 ± 1.1	4.4 ± 1.2	0.64
**Gender**			
Male	14	12	n.a.
Female	9	10	n.a.
**Dogs per musher**			
Musher A	13	15	n.a.
Musher B	5	2	n.a.
Musher C	5	5	n.a.
**Dogs per team**			
Team 1	4	4	n.a.
Team 2	5	5	n.a.
Team 3	5	2	n.a.
Team 4	4	6	n.a.
Team 5	5	5	n.a.
Plasma cortisol (ng/ml) mean ± SD	29.1 ± 11.6	29.9 ± 11.6	0.81
Plasma gastrin (pg/ml) mean ± SD	18.6 ± 5.0	17.2 ± 4.8	0.33
Plasma Ln GRP (ng/ml) mean ± SD	0.45 ± 0.54	0.26 ± 0.22	0.11
Plasma Ln CK (U/L) mean ± SD	4.1 ± 0.55	4.1 ± 0.68	0.94
Plasma lactate (mmol/l) mean ± SD	1.4 ± 0.46	1.4 ± 0.56	0.99
Leucocytes (G/L) mean ± SD	17.5 ± 1.3	17.0 ± 1.8	0.44
Hemoglobin (G/L) mean ± SD	9.7 ± 2.5	9.1 ± 2.3	0.26

*Ln, logarithmic values; SD, standard deviation*.

Creatine kinase increased significantly (*p* = 0.026) after performance, not reaching baseline within 120 min after performance (mean CK before: 77.8 ± 95.0 U/L; mean CK after: 183.9 ± 171.0 U/L; mean CK 120 min after: 220.7 ± 202.0 U/L). CK increased by 2.8-fold comparing the values before and at 120 min after exercise. CK was not normally distributed, therefore CK was logarithmically transformed for statistical analysis. There was no significant difference in ln CK comparing both treatment groups (*p* = 0.94). A significant increase of lactate (*p* = 0.014) was seen in both groups (mean lactate before: 1.4 ± 0.5 mmol/L, mean lactate after: 1.6 ± 0.3 mmol/L; mean lactate 120 min after: 0.8 ± 0.3 mmol/L). Lactate in both treatment groups was not significantly different (*p* = 0.99).

### Effect of Nx4 on Plasma Cortisol and Plasma Gastrin

**Figure 2:**
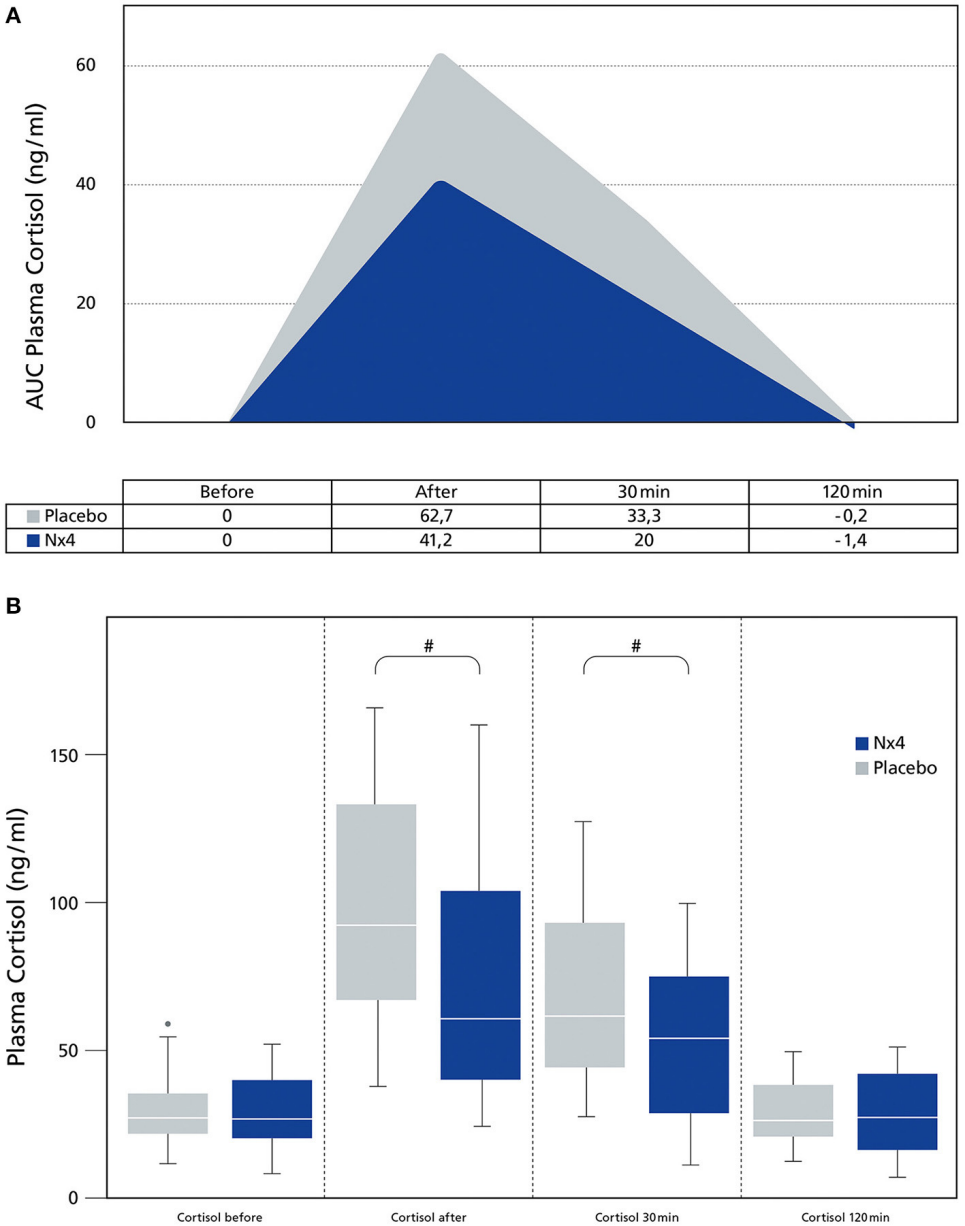
Comparing plasma Cortisol (ng/ml) between dogs receiving Nx4 or Placebo at different time points. **(A)** The data (*n* = 45), with 23 dogs in the Nx4 group and 22 dogs in the placebo group are displayed as boxplot showing the first (25%), the median (50%), and the third quartile (75%). The whiskers are displaying values within 1.5 times the IQR. Outliers are plotted as individual points. ^#^*p* = 0.038, ^##^*p* = 0.047. **(B)** The data (*n* = 45), with 23 dogs in the Nx4 group and 22 dogs in the placebo group are displayed as area under the curve. The area under the curve is the integral of a plot of the plasma cortisol (ng/ml) concentration against time. Since the baseline value (before exercise) had a significant influence, baseline was set to zero.

In this study, plasma cortisol was significantly reduced in the Nx4 group compared to the placebo group directly after performance (*p* = 0.038) and 30 min after performance (*p* = 0.047). At the 120 min time point, plasma cortisol went back to baseline in both groups (Figure 2A).

Consequently, the mean area under the curve (AUC) for plasma cortisol, adjusted for the statistically significant effect of the basal value (plasma cortisol before exercise) (*p* <0.0001) by ANCOVA, was significantly lower in the Nx4 group compared to the placebo group (*p* = 0.031) (Figure 2B).

**Figure 3:**
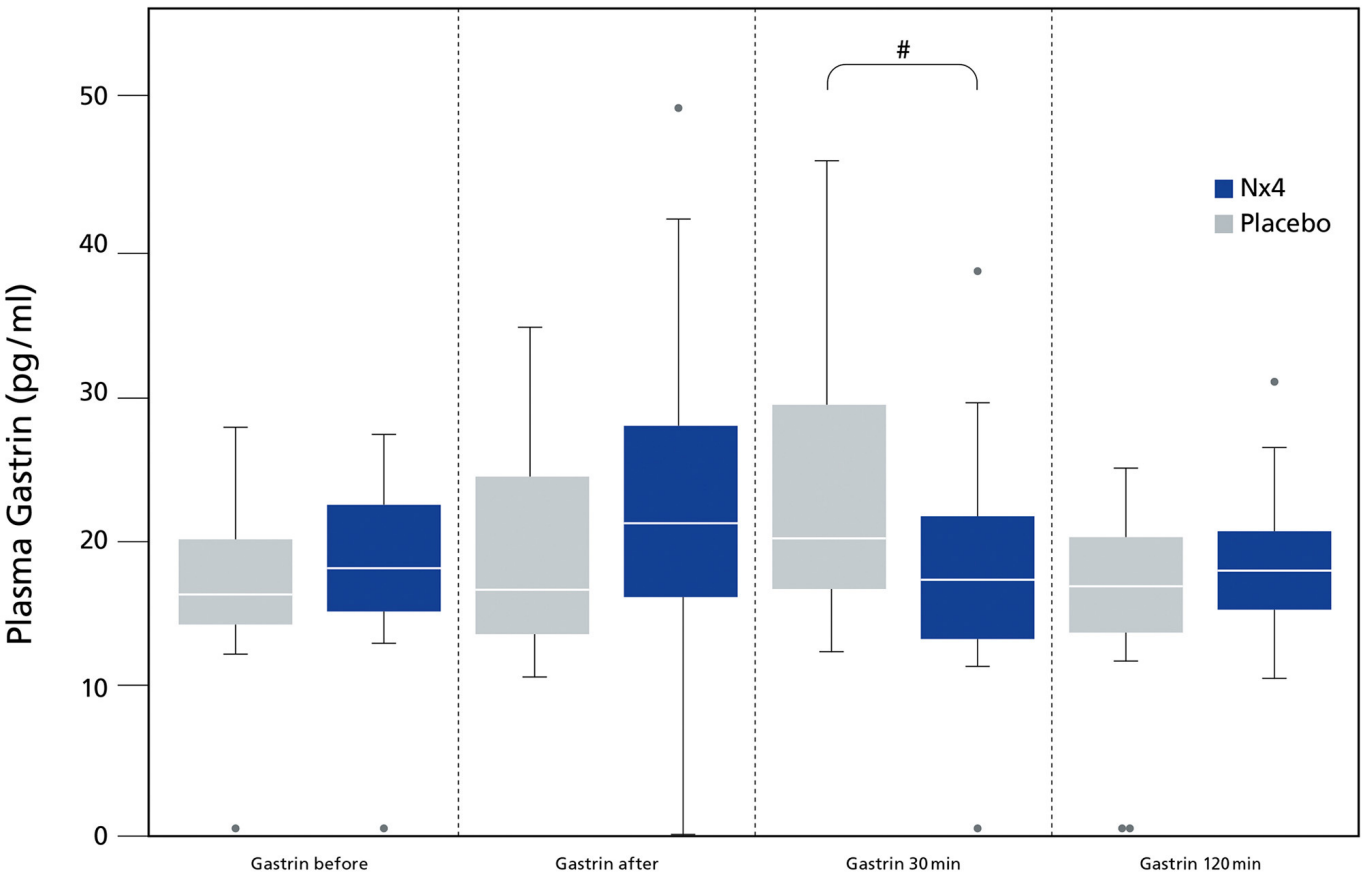
Comparing plasma gastrin (pg/ml) between dogs receiving Nx4 or Placebo at different time points. The data (*n* = 45), with 23 dogs in the Nx4 group and 22 dogs in the placebo group are displayed as boxplot showing the first (25%), the median (50%), and the third quartile (75%). The whiskers are displaying values within 1.5 times the IQR. Outliers are plotted as individual points. ^#^*p* = 0.023.

Plasma gastrin was significantly reduced in the Nx4 group 30 min after performance (*p* = 0.023) (Figure 3), resulting in a significantly reduced AUC in the Nx4 group compared to the placebo group (*p* = 0.049).

### Comparing the Pilot and the Main Study

As side analysis the values of interest of both training seasons [pilot study: beginning of the training season (2018); main study: middle of the training season (2019)] were compared. Dogs completing the pilot study and being assigned to the placebo group in the main study were used for this analysis. A subset of 10 dogs fulfilled these criteria.

Baseline plasma cortisol (plasma cortisol before exercise) increased significantly from the beginning of the training season (2018) to the middle of the training season in 2019 (*p* = 0.0034) (Supplementary Figure 1).

In concordance, repetitive exercise induced stress response caused a significant increase of baseline plasma gastrin (plasma gastrin before exercise: *p* <0.0001) from the beginning of the training season to the middle of the training season (Supplementary Figure 2).

To elucidate, if chronic repetitive stress can cause a decrease in total leucocyte count, the two groups were compared and a significant (*p* = 0.013) reduction of leucocytes from the beginning to the middle of the training season was found (Supplementary Figure 3).

## Discussion

**Figure 4:**
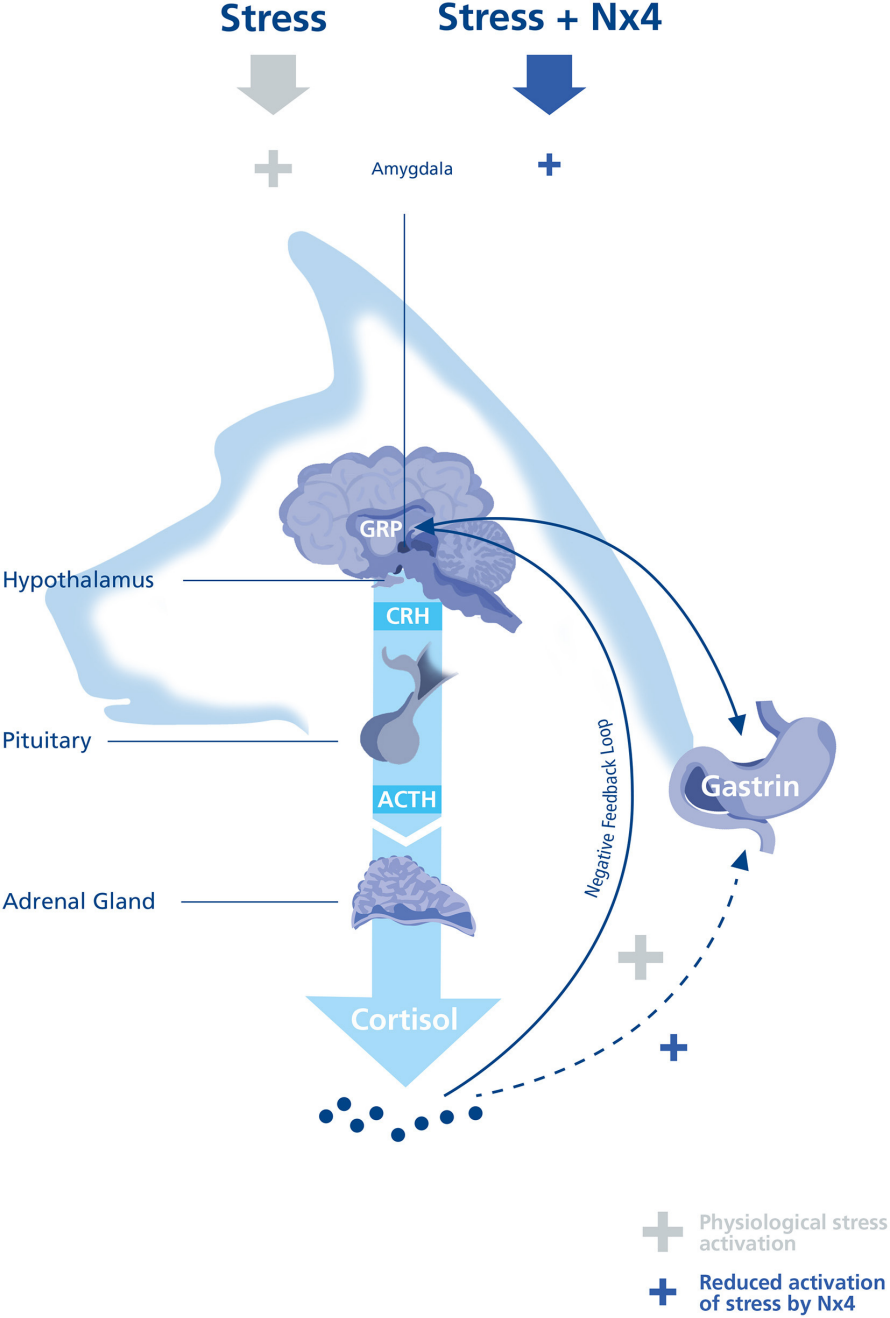
Illustration of the HPA axis in a dog. Hypothalamic-pituitary-adrenal (HPA) axis activation is dependent on the stressor duration and intensity. A typical stress response begins with the release of Corticotropin releasing hormone (CRH) from the Hypothalamus causing adrenocorticotropic hormone (ACTH) from the pituitary to induce cortisol release. The time course of cortisol increase is depending on a combination of the activation of the negative feedback loop and by degradation of cortisol. Under physiological conditions gastrin releasing peptide (GRP) is responsible for Gastrin release to induce the gastric acid and pepsin production. Under exercise induced stress, it seems possible that Gastrin release can be activated directly or indirectly by cortisol.

Assessing exercise induced stress response, it is important to note that the HPA axis is one of many endogenous stress-reactive systems (8). In this study, we focused on plasma cortisol as a well-established biomarker for exercise induced stress in sled dogs during performance. Cortisol is an indirect marker of psychological and exercise induced stress, being an effector glucocorticoid of the HPA axis (Figure 4).

In general, activation of the HPA axis is engendered by all varieties of stressor, and is a hallmark of the physiological reaction to stress (8). While, other markers beside plasma cortisol might have been of interest, many catecholamines, and cytokines involved in an exercise induced stress response are of very short half-life. This is also true for ACTH, and corticotropin-releasing hormone (CRH) (7), and under field conditions these parameters are difficult to study.

The pilot study confirmed a significant increase of plasma cortisol in a single exercise induced stress setting (*p* = 0.001, Figure 1), covering a relatively short distance of ~25 km with untrained dogs after the summer break. The duration and intensity of the training was enough to exceed the threshold for an exercise induced stress response. Exercise induced increase of serum cortisol was also seen in previous studies in sled dogs after performance (1–3, 30). Cortisol release is needed during exercise (31), but increased cortisol can cause negative side effects such as gastritis, gastric ulcer, hypoglycemia and immunosuppression (12, 32, 33). Signs of gastritis and gastric ulcers are abdominal pain, anorexia and vomiting (34), affecting the welfare of sled dogs.

Nx4 significantly decreased plasma cortisol (AUC, *p* = 0.031, Figure 2B) during a single acute exercise session in sled dogs in the main study. Nx4 has previously been studied in human medicine showing a reduction of blood cortisol during an acute stress setting (20) comparable to the reduction in plasma cortisol measured in this study. The mode of action of Nx4 seems to be mediated by a reduced activation of the amygdala (23) and an alteration of the delta- and theta-waves in the EEG of rats (19) and an alteration of the EEG in humans (24). The reduced activation of the amygdala can result in a reduced activity of the HPA-axis, resulting in decreased cortisol levels.

If measuring cortisol, many influencing factors must be considered to reduce the chance of measuring artificial values. This study tried to control for some of the known influencing factors (5). The circadian rhythm is one of these factors. Cortisol shows a peak in the morning just before waking up, then cortisol decreases gradually during the day (35, 36). To receive comparable results, a rigid time schedule was followed each day (Figure 1). Additionally, exercise duration (180 min) and intensity (distance: 35 km) were the same for all dogs, confirmed by comparable values of lactate and CK in both treatment groups. Lactate is an indicator of glycolytic activity of the skeletal muscle and is often used as marker for the intensity of exercise and the fitness of the individual (37).

The training schedule before the start of the main study was kept comparable for all dogs and all dogs were trained every other day. Dogs did not show different baseline plasma cortisol levels between mushers or teams (Table 2), indicating that the impact of slightly different training schedules did not influence the results. All dogs participating in the study received the same diet to control for intake of macronutrients (5). Additionally, it is also known that dogs are very susceptible to the stress of the owner (38). Sled dogs tend to have a looser bond to their owner, believing that this influencing factor should not have a strong impact on the obtained values, even so an influence cannot be ruled out completely. The temperature can have an influence on plasma cortisol levels (5), and during the 5 days of the study slightly different temperatures could not be prevented and could have had an influence on plasma cortisol values.

Gastrin is a peptide hormone which is involved in the process of gastric acid secretion. Gastrin release is induced by GRP, a neurotransmitter acting on its basolateral receptor in G-cells, resulting in gastric acid production (39).

In our study, plasma gastrin was significantly lowered 30 min after performance (*p* = 0.023, Figure 3) in dogs receiving Nx4 compared to dogs receiving placebo. Our study did not find differences in GRP in both treatment groups, raising the question if cortisol can mediate directly or indirectly gastrin release. Increased gastrin levels have been seen after long distance runs and during short recreational mushing in sled dogs and were accompanied by increased cortisol (1–3, 18). In humans with hypercortisolism or chronic prednisolone administration increased gastrin values can be seen (33, 40), but Seino et al. did not see increased gastrin values after a single infusion of cortisol (40). This finding is in accordance to our pilot study on untrained dogs were gastrin values were low and showed no increase by performance. On the other hand, a significant increase of gastrin was seen after a single infusion of cortisol in patients with chronic prednisolone treatment (40). Dogs in the main study showed a significant increase in baseline cortisol comparable to chronic low dose prednisolone treatment and this finding might explain the increase of gastrin after performance in the dogs in the main study (Figure 4).

To understand the different findings of plasma gastrin comparing the pilot and the main study we decided to do a sub analysis with dogs from the pilot study, that were assigned to the placebo group in the main study (*n* = 10). This analysis was of interest to understand if repetitive exercise induced stress response by training every other day can explain the high prevalence for gastric ulcers of 41.5–61% found in racing sled dogs and in trained sled dogs (15–17). In this study, we found a significant increase (*p* <0.0001, Supplementary Figure 2) in plasma gastrin comparing the pilot (at the beginning of the training season, 2018) and the main study conducted in the middle of the training season (2019). Plasma cortisol was also significantly increased (*p* = 0.0034, Supplementary Figure 1) in the main study compared to the pilot study. The increase of baseline cortisol might show that repetitive exercise during a training season can set a new point of baseline cortisol as an adaptation to the repetitive exercise induced stress (10, 11). This is also seen in human endurance athletes were repetitive strenuous exercise leads to increased basal glucocorticoid levels comparable to this cohort of dogs (31). If baseline plasma cortisol is increased in the middle of the training season, this might reflect patients with chronic prednisolone treatment showing significantly increased gastrin levels after a single infusion of cortisol (40). If the gastrin induction is mediated by cortisol, this could explain the delayed reduction of gastrin by Nx4 30 min after cortisol was reduced (Figure 4).

On one hand this finding is of interest, because it is believed that high gastrin levels might cause gastric ulcers, affecting the welfare of sled dogs. On the other hand, there is still a significant controversy, if a rise in plasma gastrin is responsible for the development of gastritis and gastric ulcers in sled dogs. It is well-established, that gastric ulcers develop in trained dogs under racing stress, and gastritis and gastric ulcers were also seen in trained but not racing dogs (17). Several studies demonstrated a significant reduction of exercise induced gastritis and gastric ulcers by gastric acid reducing drugs, suggesting a significant impact of gastric acid on the development of this disease (41–43).

Limitations of our results are that GRP was only measured in the main study before and after performance, but it is possible, that GRP increased during performance, going back to baseline before end of performance. Another limitation was that the dogs were fed 1 h prior to the first blood sampling and 3.5 h before performance, but this was the same for dogs in the pilot and in the main study and for dogs assigned to the Nx4 or the placebo group. The decision to feed the dogs in the morning was made based on the normal daily routine of waking up and feeding the dogs and believing that not feeding the dogs would cause psychological stress, by not following their normal routine.

Other limitating factors of the sub analysis were the time interval between the pilot and the main study and the low number of dogs (*n* = 10) in this subgroup.

A limitation of our study was also the used acute dosing regimen comparable to the study from Doering et al. (20). This dose is recommended in the case of an upcoming stressful event. Further studies are needed to elucidate if a lower dose would also have a comparable effect on plasma cortisol and plasma gastrin as seen in the study from Herrman et al. were a single dose was sufficient to reduce the activity of the amygdala (23).

The second aim of the main study was to assess tolerability of Nx4. No acute side effects, for example nervousness, have been observed by the musher or the study personnel after the single high dose used in this study, showing that Nx4 was well-tolerated in this specific setting. Further studies are needed to elucidate the question if Nx4 is also safe when used more frequently and the scope of the study did not allow to obtain toxicological data for Nx4.

## Conclusion

Within the limitation of the study, the results carry implications for the usefulness of Nx4 to reduce stress-induced plasma cortisol and gastrin levels. The reduction of the stress response could help to improve the welfare of high-performance sled dogs, but further studies are needed to answer this question. Since stress induced increase of cortisol is similar for physical and psychologic stress, the same might be true independent of the stressor, making Nx4 useful in any stressful situation for dogs.

## Data Availability Statement

The original contributions presented in the study are included in the article/Supplementary Material, further inquiries can be directed to the corresponding author/s.

## Ethics Statement

The animal study was reviewed and approved by Norwegian Food and Safety Authority (FOTS ID 17660) based on the use of animals for scientific purposes (forskrift 18. juni 2015 nr. 781om bruk av dyr i forsøk). Written informed consent was obtained from the owners for the participation of their animals in this study.

## Author Contributions

AK and MW: study design and decision to publish. AK, JC, CW, KF, and MW: data collection, analysis, review, and editing of the manuscript. AK: preparation of the manuscript. All authors contributed to the article and approved the submitted version.

## Funding

The study was solely funded by Heel GmbH, Baden-Baden, Germany, which employed the author MW. The funder provided support in form of financial compensation for AK, JC, CR, and KF and the salary for the author MW. The funder provided the test substance and placebo and was involved in practical implementation, data collection, analysis, and decision to publish.

## Conflict of Interest

MW is employed by Heel GmbH, Baden-Baden, Germany, who funded this study. All remaining author received a financial compensation from Heel GmbH, Baden-Baden, Germany in form of travel reimbursement and consulting fees. JC was employed by Dartsch Scientific GmbH. CW was employed by Laboklin GmbH & Co. KG. The remaining authors declare that the research was conducted in the absence of any commercial or financial relationships that could be construed as a potential conflict of interest.

## Publisher's Note

All claims expressed in this article are solely those of the authors and do not necessarily represent those of their affiliated organizations, or those of the publisher, the editors and the reviewers. Any product that may be evaluated in this article, or claim that may be made by its manufacturer, is not guaranteed or endorsed by the publisher.

## Supplementary Material

Supplementary Figure 1 Comparing plasma Cortisol levels (ng/ml) from the start of the training season to the middle of the training season. The data (*n* = 10) are displayed as boxplot showing the first (25%), the median (50%), and the third quartile (75%). The whiskers are displaying values within 1.5 times the IQR. Outliers are plotted as individual points. ^#^*p* = 0.0034.

Supplementary Figure 2 Comparing plasma gastrin levels (pg/ml) from the start of the training season to the middle of the training season. The data (*n* = 10) are displayed as boxplot showing the first (25%), the median (50%), and the third quartile (75%). The whiskers are displaying values within 1.5 times the IQR. Outliers are plotted as individual points. ^#^*p* <0.0001.

Supplementary Figure 3 Comparing leucocytes (G/L) from the start of the training season to the middle of the training season. The data (*n* = 10) are displayed as boxplot showing the first (25%), the median (50%), and the third quartile (75%). The whiskers are displaying values within 1.5 times the IQR. Outliers are plotted as individual points. ^#^*p* = 0.013.
