# Supplementary figures and images for: Efficacy of Nx4 to Reduce Plasma Cortisol and Gastrin Levels in Norwegian Sled Dogs During an Exercise Induced Stress Response: A Prospective, Randomized, Double Blinded, Placebo-Controlled Cohort Study
Source: Front Vet Sci. 2021 Oct 26;8:741459. doi: 10.3389/fvets.2021.741459 (PMC8576320; doi:10.3389/fvets.2021.741459)

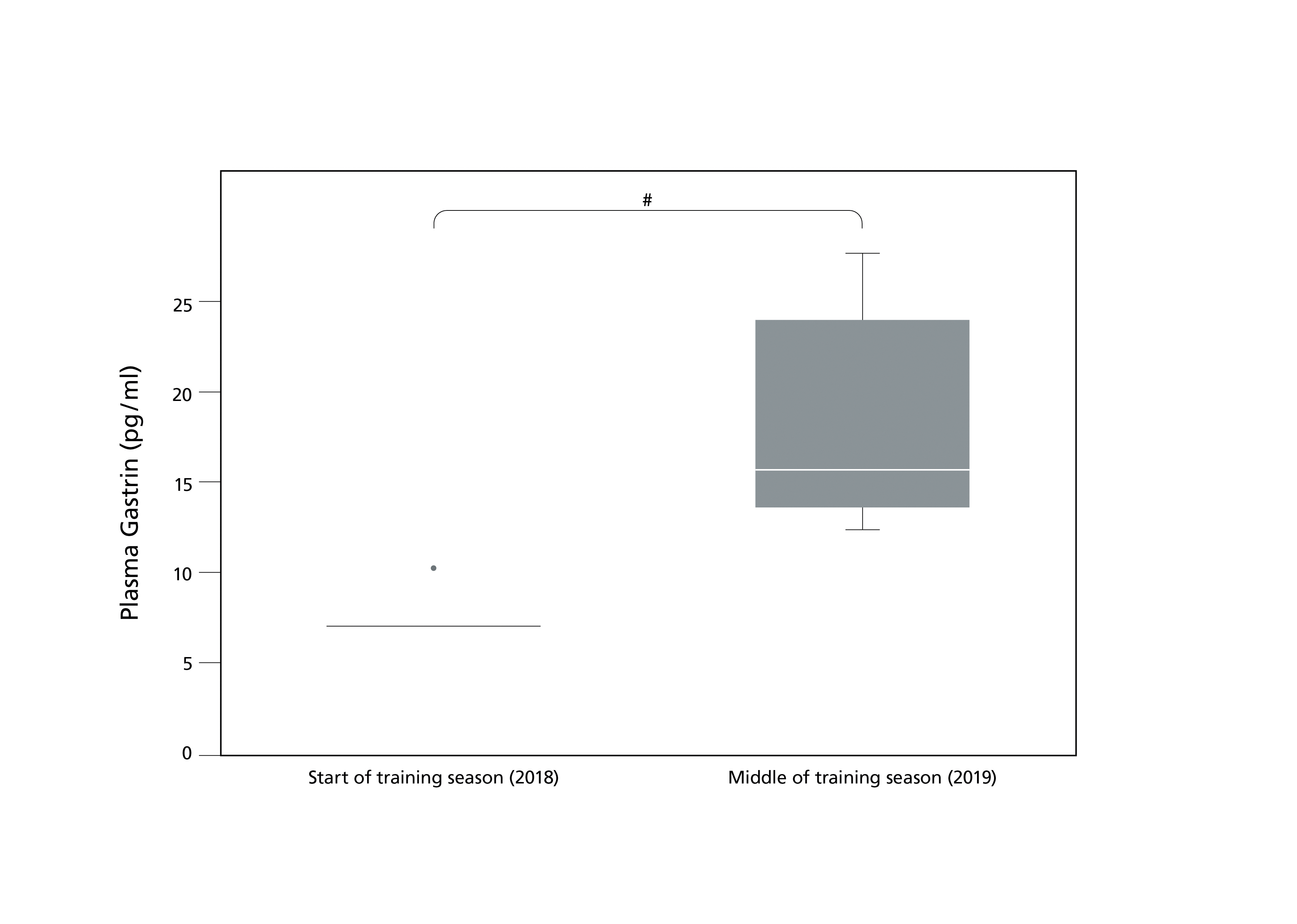

Supplement: Supplementary Figure 1 — Comparing plasma Cortisol levels (ng/ml) from the start of the training season to the middle of the training season. The data (n = 10) are displayed as boxplot showing the first (25%), the median (50%), and the third quartile (75%). The whiskers are displaying values within 1.5 times the IQR. Outliers are plotted as individual points. #p = 0.0034. [file Image_1.TIF]

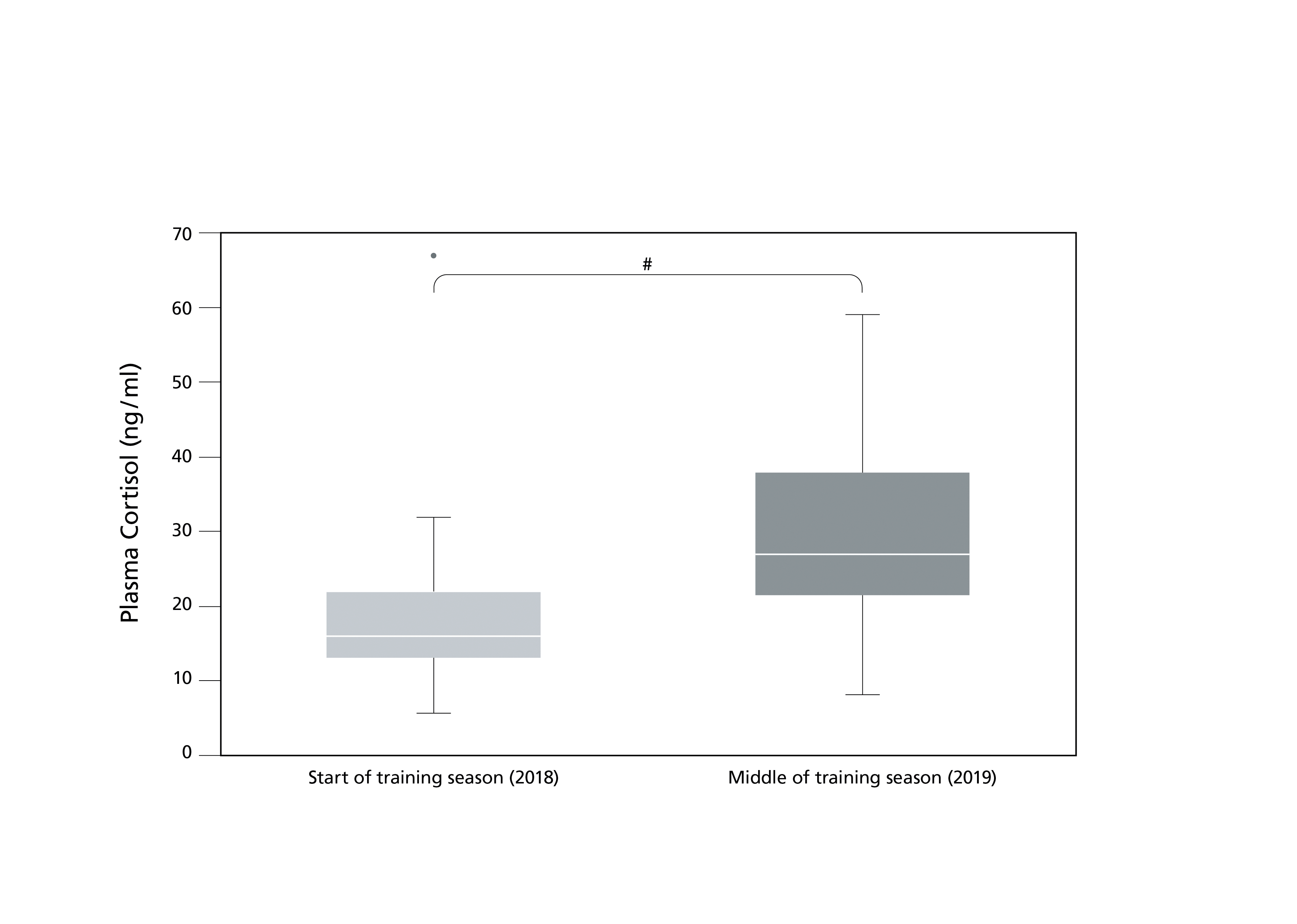

Supplement: Supplementary Figure 2 — Comparing plasma gastrin levels (pg/ml) from the start of the training season to the middle of the training season. The data (n = 10) are displayed as boxplot showing the first (25%), the median (50%), and the third quartile (75%). The whiskers are displaying values within 1.5 times the IQR. Outliers are plotted as individual points. #p <0.0001. [file Image_2.TIF]

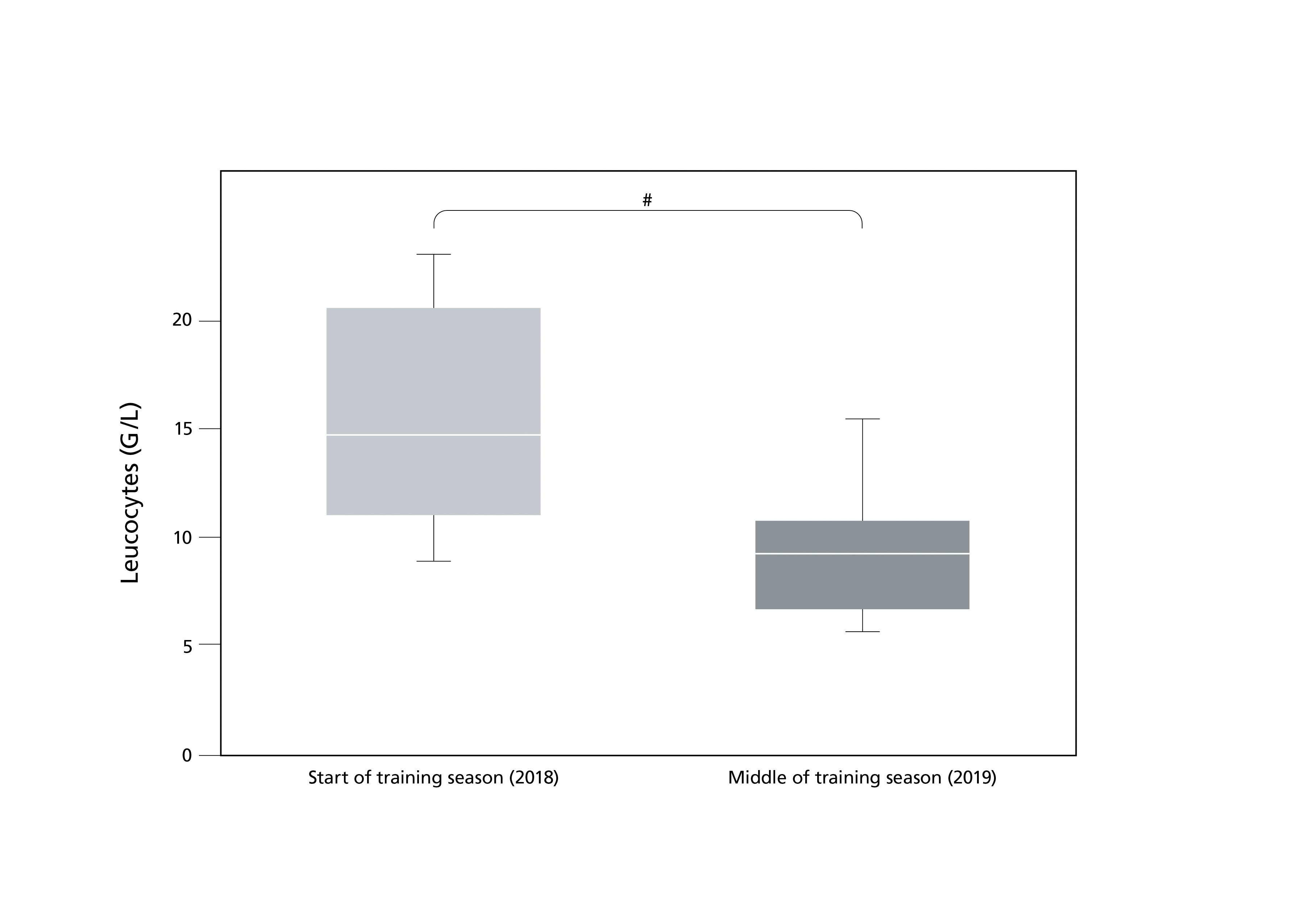

Supplement: Supplementary Figure 3 — Comparing leucocytes (G/L) from the start of the training season to the middle of the training season. The data (n = 10) are displayed as boxplot showing the first (25%), the median (50%), and the third quartile (75%). The whiskers are displaying values within 1.5 times the IQR. Outliers are plotted as individual points. #p = 0.013. [file Image_3.TIF]
